# Deletion of *luxI* increases luminescence of *Vibrio fischeri*

**DOI:** 10.1128/mbio.02446-24

**Published:** 2024-09-24

**Authors:** Kathryn A. Bellissimo, Alecia N. Septer, Cheryl A. Whistler, Coralis Rodríguez, Eric V. Stabb

**Affiliations:** 1 Department of Microbiology, University of Georgia, Athens, Georgia, USA; 2 Department of Biological Sciences, University of Illinois at Chicago, Chicago, Illinois, USA; 3 Department of Earth, Marine & Environmental Sciences, University of North Carolina, Chapel Hill, North Carolina, USA; 4 Department of Molecular, Cellular, and Biomedical Sciences, University of New Hampshire, Durham, New Hampshire, USA; Georgia Institute of Technology, Atlanta, Georgia, USA

**Keywords:** *Photobacterium*, *Aliivibrio*, quorum sensing, bioluminescence

## Abstract

Bioluminescence in *Vibrio fischeri* is regulated by a quorum-dependent signaling system composed of LuxI and LuxR. LuxI generates *N-*3-oxohexanoyl homoserine lactone (3OC6-HSL), which triggers LuxR to activate transcription of the *luxICDABEG* operon responsible for bioluminescence. Surprisingly, a ∆*luxI* mutant produced more bioluminescence than the wild type in culture. In contrast, a 4 bp duplication within *luxI*, resulting in a frameshift mutation and null allele, decreased luminescence tenfold. A second signaling system encoded by *ainSR* affects bioluminescence by increasing levels of LuxR, via the transcriptional activator LitR, and the *N-*octanoyl homoserine lactone (C8-HSL) signal produced by AinS is considered only a weak activator of LuxR. However, *ainS* is required for the bright phenotype of the ∆*luxI* mutant in culture. When 3OC6-HSL was provided either in the medium or by expression of *luxI in trans*, all cultures were brighter, but the ∆*luxI* mutant remained significantly brighter than the *luxI* frameshift mutant. Taken together, these data suggest that the enhanced bioluminescence due to the LuxI product 3OC6-HSL counteracts a negative *cis*-acting regulatory element within the *luxI* gene and that when *luxI* is absent the C8-HSL signal is sufficient to induce luminescence.

**IMPORTANCE:**

The regulation of bioluminescence by *Vibrio fischeri* is a textbook example of bacterial quorum-dependent pheromone signaling. The canonical regulatory model is that an autoinducer pheromone produced by LuxI accumulates as cells achieve a high density, and this LuxI-generated signal stimulates LuxR to activate transcription of the lux operon that underlies bioluminescence. The surprising observation that LuxI is dispensable for inducing bioluminescence forces a re-evaluation of the role of *luxI*. More broadly, the results underscore the potential deceptiveness of complex regulatory circuits, particularly those in which bacteria produce multiple related signaling molecules.

## OBSERVATION

Regulation of bioluminescence in *Vibrio fischeri* is a long-standing model of cell density (or “quorum”)-dependent behaviors (1), and this phenomenon is attributed to regulation embedded in the *lux* locus (2). The *lux* genes responsible for bioluminescence are co-transcribed with *luxI*, forming the *luxICDABEG* operon, which is divergently transcribed from *luxR*. LuxI synthesizes *N-*3-oxohexanoyl homoserine lactone (3OC6-HSL) (3), which diffuses through membranes and acts as a pheromone signal (4). Upon reaching a critical concentration, 3OC6-HSL combines with LuxR and stimulates transcription of *luxICDABEG*, thereby generating bioluminescence and, via positive feedback typical of such signaling (5), more 3OC6-HSL. Interestingly, bioluminescence is a colonization factor for *V. fischeri* in its symbiosis within the light-emitting organ of the Hawaiian bobtail squid, *Euprymna scolopes*. Specifically, *lux* mutants lacking bioluminescence initially colonize the host but do not persist well for unknown reasons (6–9). Both *luxI* and *luxR* mutants produced little or no bioluminescence during host colonization, supporting the idea that LuxI/LuxR-mediated regulation underlies symbiotic bioluminescence (8, 10).

The *ainSR* locus in *V. fischeri* encodes a second acyl-homoserine lactone signaling system, which is thought to function primarily by priming the *lux* system at lower cell densities (10–13). AinS generates the pheromone *N-*octanoyl homoserine lactone (C8-HSL), but in contrast to the chemical similarity of the Lux and Ain signals, AinS and AinR are structurally unrelated to LuxI and LuxR, and they function mainly through a distinct regulatory cascade. AinR is a receptor for C8-HSL, and it influences bioluminescence via LitR, which activates the transcription of *luxR* (14). C8-HSL can activate LuxR directly, although this effect appears to be weak (15). The model that has emerged is that the Ain system primes the Lux system in two ways (10): (i) the combination of C8-HSL and AinR leads to more LitR and consequently more LuxR; and (ii) C8-HSL combines with LuxR, and together they weakly activate *luxICDABEG*, engaging LuxI-mediated positive feedback and higher 3OC6-HSL production. Beyond this priming role, C8-HSL can interfere with 3OC6-HSL-mediated activation of luminescence (15), and *ainS* mutants can be brighter than wild type in colonies on solid media (16). The addition of C8-HSL or 3OC6-HSL to *ainS* and *luxI* mutants, respectively, restored luminescence phenotypes (10), indicating that the signal synthase activity of AinS and LuxI is critical to their regulatory roles. Given what is known, LuxI and 3OC6-HSL are considered essential for robust induction of luminescence.

**FIG 1:**
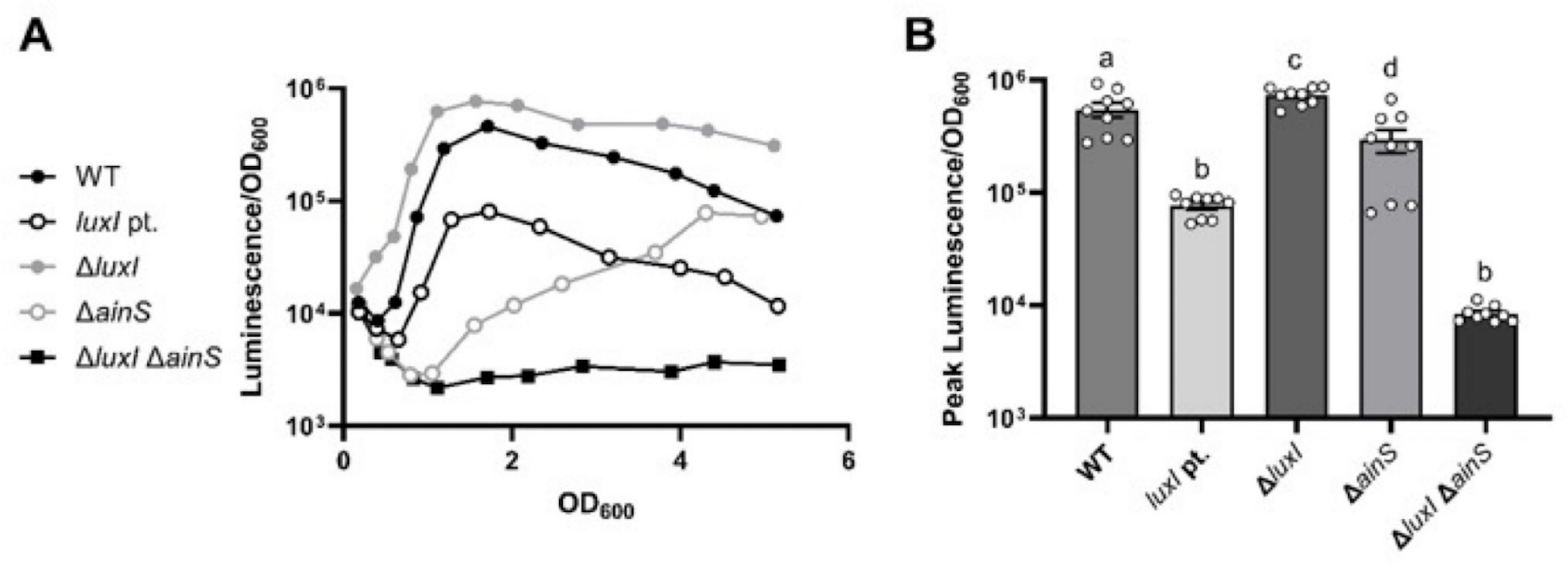
The *luxI* gene is not necessary for luminescence induction. (A) Specific luminescence as a function of cell density for ES114 wild type (WT, black circles), *luxI* point mutant VCW2G7 (*luxI* pt., open black circles), *luxI* deletion mutant, ANS3 (Δ*luxI*, gray circles), *ainS* deletion mutant, NL60 (Δ*ainS*, open gray circles), and *luxI ainS* double deletion mutant, KB12 (Δ*luxI* Δ*ainS*, black squares). (B) Peak luminescence values (Luminescence/OD_600_) for indicated strains. To determine statistically significant differences between treatments in panel (B), a one-way ANOVA was performed, followed by a multiple *t*-test with Fisher’s least significant difference (LSD) post-test. Letters indicate statistical relatedness (*P* < 0.05). Error bars indicate standard error (*n* = 9). Panels (A) and (B) show representatives of multiple experiments performed at least three times.

Based on this prevailing model, we were surprised to observe that the ∆*luxI* mutant ANS3 produced more light in culture than the wild-type parent ES114 (Fig. 1). Although ANS3 was previously used to generate 3OC6-HSL-free culture supernatant (17), this is the first report of its unexpected luminescence phenotype. In contrast to ANS3, mutant VCW2G7 (10), a frameshift mutant that has a 4 bp duplication near the 3′ end of *luxI*, was dimmer than ES114 (Fig. 1), as previously reported (10). Both *luxI* mutants displayed density-dependent luminescence induction (Fig. 1A), suggesting accumulation of C8-HSL triggers luminescence. Consistent with this model, the Δ*ainS* Δ*luxI* double-mutant KB12 was dim and did not induce luminescence (Fig. 1), indicating that in the absence of *luxI* bright luminescence requires *ainS*, which encodes the C8-HSL synthase.

**FIG 2:**
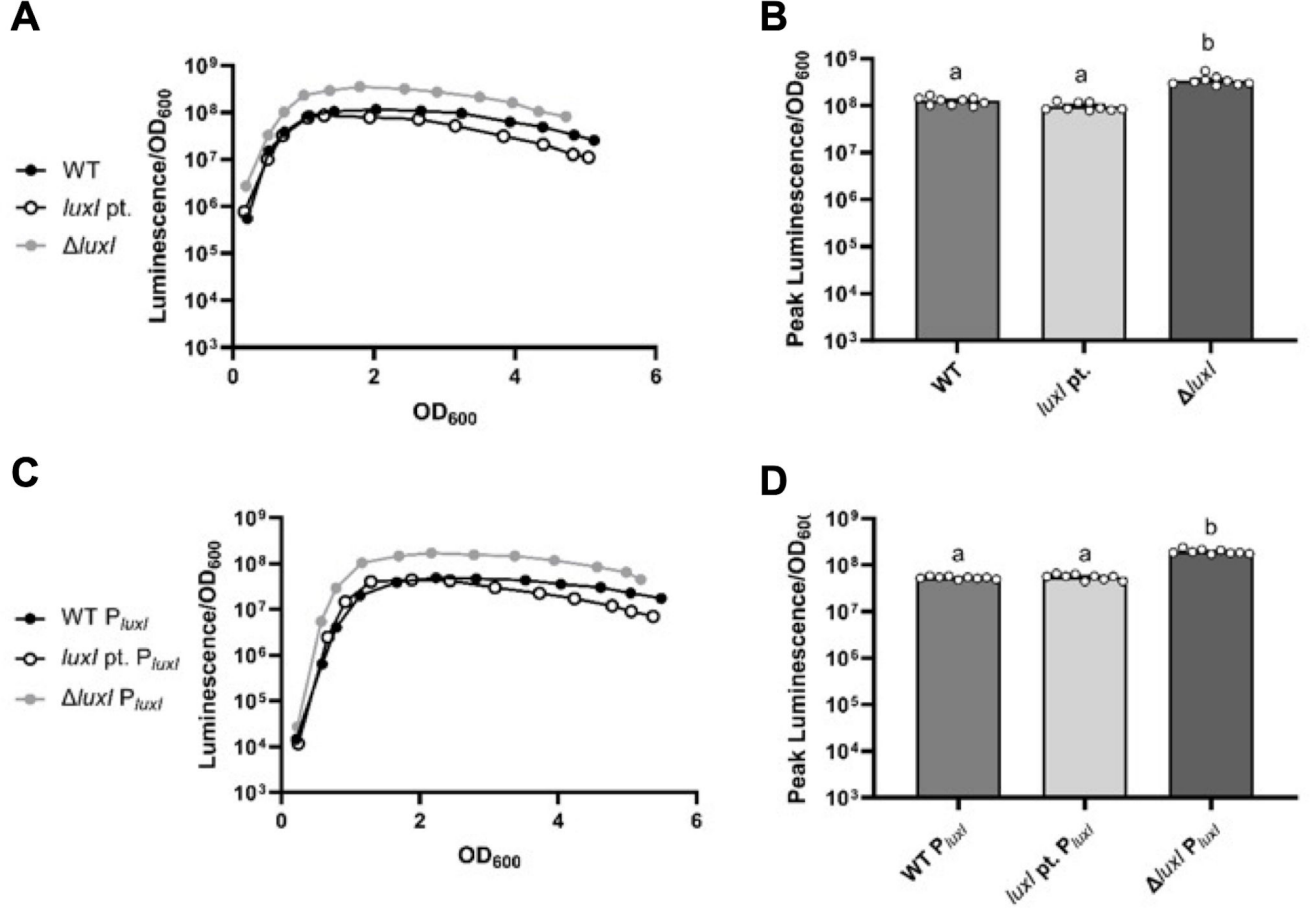
Effect of exogenous 3OC6-HSL or *luxI in trans* on *luxI* mutants and wild type. (A and C) Specific luminescence as a function of cell density for the following strains grown with 3200 nM 3OC6-HSL added exogenously (A) or with *luxI* expressed *in trans* from plasmid pCRG36 (C): ES114 wild type (WT, black circles), *luxI* point mutant VCW2G7 (*luxI* pt., open black circles), and *luxI* deletion mutant ANS3 (Δ*luxI*, gray circles). (B and D) Peak luminescence values (Luminescence/OD_600_) for the indicated strains with added 3OC6-HSL (B) or carrying *luxI* on pCRG36 (D). Induction of *luxI* on pCRG36 was achieved by adding 500 µM isopropyl-ß-d-thiogalactoside. To determine statistically significant differences between treatments in panel (B), an ANOVA was performed, followed by a multiple *t*-test with Fisher’s LSD post-test. Letters indicate statistical relatedness (*P* < 0.05). Error bars indicate standard error (*n* = 9). Panels show representatives of multiple experiments performed at least three times. 3OC6-HSL, *N-*3-oxohexanoyl homoserine lactone.

The different luminescence phenotypes of ANS3 and VCW2G7 presumably reflect their different *luxI* mutations. The ∆*luxI* allele in ANS3 represents a deletion of the entire gene with the insertion of 5′-GCTAGC-3′ (an NheI restriction site) between the *luxI* start and stop codons. VCW2G7, on the other hand, was generated by cleaving a BglII site toward the 3′ end of *luxI*, filling in the overhang, and re-ligating to produce a 4 bp insertion, leading to a non-functional allele. Thus, virtually the entire *luxI* sequence is absent in ANS3 but present in VCW2G7. We further tested the effects of these *luxI* mutant alleles in the presence of 3OC6-HSL added exogenously (Fig. 2A and B) or produced by expressing *luxI in trans* from pCRG36 (Fig. 2C and D). The addition of 3OC6-HSL or *in trans* expression of *luxI* increased luminescence for ES114 and the two *luxI* mutants, but the *luxI* point mutant achieved the same level of luminescence as ES114, whereas the ∆*luxI* mutant was significantly brighter (*P* < 0.05) than the other two strains (Fig. 2).

The observations reported here upend the canonical regulatory model that 3OC6-HSL produced by LuxI is required for robust induction of bioluminescence in *V. fischeri*. This bacterium has been studied intensively, particularly as a model for bacterial quorum-dependent pheromone signaling and for its symbioses (18–21), yet our results encourage a re-evaluation of the core regulatory circuit governing bioluminescence. Under the conditions tested here, the positive regulatory effect of 3OC6-HSL seems to counterbalance partially a negative regulatory effect lost in a ∆*luxI* mutant. We propose this negative regulatory element is *cis*-acting and embedded within the *luxI* gene or mRNA, such that the *luxICDABEG* operon is negatively regulated by a mechanism that does not act on *luxCDABEG* when *luxI* is deleted from the operon and provided *in trans*. Absent these counteracting forces of the *luxI* sequence and the LuxI product, C8-HSL is able to induce luminescence more than we anticipated based on earlier studies. Future work should help elucidate the mechanisms behind these unexpected observations.

## METHODS

Table S1 describes oligonucleotides, plasmids, strains, and their construction. *V. fischeri* was grown in lysogeny broth salts (LBS) medium (22) at 28°C, except for luminescence experiments in seawater tryptone osmolarity (SWTO) medium at 24°C (23). We used established conditions for growing *Escherichia coli* and antibiotic selection (24).

## SUPPLEMENTAL MATERIAL

Table S1 mbio.02446-24-s0001.pdf Strains, plasmids, oligonucleotides, and corresponding references.

ASM does not own the copyrights to Supplemental Material that may be linked to, or accessed through, an article. The authors have granted ASM a non-exclusive, world-wide license to publish the Supplemental Material files. Please contact the corresponding author directly for reuse.
