# Supplementary material for: Deletion of luxI increases luminescence of Vibrio fischeri
Source: mBio. 2024 Sep 24;15(10):e02446-24. doi: 10.1128/mbio.02446-24 (PMC11481858; doi:10.1128/mbio.02446-24)
Supplement: Table S1 — Strains, plasmids, oligonucleotides, and corresponding references. [file mbio.02446-24-s0001.pdf]

**Supplemental Table S1.** Select Strains and Plasmids

| Strains or Plasmids | Relevant characteristics[a] | Source or Reference |
|---|---|---|
| ***E. coli*** | | |
| DH5α | F'/*endA1 hsdR17 glnV44 thi-1 recA1 gyrA relA1* Δ(*lacIZYA-argF*)*U169deoR*(f80*dlacI*Δ(*lacZ*)*M15*) | (Hanahan 1983) |
| CC118λpir | Δ(*ara-leu*) *araD* Δ*lac74 galE galK phoA20 thi-1 rpsE rpsB argE*(Am) *recA λpir* | (Herrero, de Lorenzo, and Timmis 1990) |
| ***V. fischeri*** | | |
| ES114 | Wild-type isolate from *Euprymna scolopes* light organ | (Boettcher and Ruby 1990) |
| ANS3 | ES114 Δ*luxI*; *luxI* 3OC6 synthase (VF_A0924) gene deletion | (Septer and Stabb 2012) |
| VCW2G7 | ES114 *luxI* point mutant | (Lupp et al. 2003) |
| NL60 | ES114 Δ*ainS*; *ainS* C8 synthase (VF_1037) gene deletion | (Lyell et al. 2013) |
| KB12 | ES114 Δ*ainS* Δ*luxI* double mutant; Δ*ainS* allele on pNL62 into AN3 | This study[b] |
| **Plasmids**[b] | | |
| pAS3 | Δ*luxI* allele; $oriV_{R6K\gamma}$, $oriV_{ColE1}$, *oriT*, $Erm^R$, $Kn^R$ | (Septer and Stabb 2012) |
| pCRG36 [c] | *luxI, lacI*$^q$, $oriV_{R6K\gamma}$, $oriV_{pES213}$, *oriT*, $Kn^R$ | This study |
| pNL62 | Δ*ainS* allele; $oriV_{R6K\gamma}$, $oriV_{ColE1}$, *oriT*, $Erm^R$, $Kn^R$ | (Lyell et al. 2013) |
| pEVS104 | conjugative helper, $oriV_{R6K\gamma}$, *oriT*, $Kn^R$ | (Stabb and Ruby 2002) |
| **Oligonucleotides (5' to 3')** [d] | | |
| CRG53 | ACT<u>GGTACC</u>TAAGAGAGGTTGCATGGCTGT | This study |
| CRG54 | ACT<u>GCTAGC</u>TTAATTTGATACAGCTTTTC | This study |

[a] $Kn^R$, Kanamycin resistance; $Erm^R$, Erythromycin resistance. Plasmid replication origins are designated *oriV* with a subscript indicating the source, and *oriT* indicates the RP4 origin of transfer.

[b] Plasmids were maintained in DH5α or in CC118λpir for pEVS104. Conjugation of plasmids used pEVS104 as a helper (Stabb and Ruby 2002).

[c] pCRG36 consisted of *luxI* PCR amplified from pJLB73 (Bose et al. 2007) using primers CRG53 and CRG54, digested with KpnI and NheI and cloned into the corresponding sites of pAKD601B (Dunn et al. 2010)

[d] Oligonucleotides are in the 5' to 3' orientation and underlined sequences highlight restriction enzyme recognition sites used for cloning

**References**:


Boettcher, K. J., and E. G. Ruby. 1990. 'Depressed light emission by symbiotic *Vibrio fischeri* of the sepiolid squid *Euprymna scolopes*', *Journal of bacteriology*, 172: 3701-6.

Bose, J. L., U. Kim, W. Bartkowski, R. P. Gunsalus, A. M. Overley, N. L. Lyell, K. L. Visick, and E. V. Stabb. 2007. 'Bioluminescence in *Vibrio fischeri* is controlled by the redox-responsive regulator ArcA', *Molecular Microbiology*, 65: 538-53.

Dunn, A. K., E. A. Karr, Y. Wang, A. R. Batton, E. G. Ruby, and E. V. Stabb. 2010. 'The alternative oxidase (AOX) gene in *Vibrio fischeri* is controlled by NsrR and upregulated in response to nitric oxide', *Molecular Microbiology*, 77: 44-55.

Hanahan, D. 1983. 'Studies on transformation of *Escherichia coli* with plasmids', *Journal of molecular biology*, 166: 557-80.

Herrero, M., V. de Lorenzo, and K. N. Timmis. 1990. 'Transposon vectors containing non-antibiotic resistance selection markers for cloning and stable chromosomal insertion of foreign genes in gram-negative bacteria', *Journal of bacteriology*, 172: 6557-67.

Lupp, C., M. Urbanowski, E. P. Greenberg, and E. G. Ruby. 2003. 'The *Vibrio fischeri* quorum-sensing systems *ain* and *lux* sequentially induce luminescence gene expression and are important for persistence in the squid host', *Molecular microbiology*, 50: 319-31.

Lyell, N. L., D. M. Colton, J. L. Bose, M. P. Tumen-Velasquez, J. H. Kimbrough, and E. V. Stabb. 2013. 'Cyclic AMP Receptor Protein Regulates Pheromone-Mediated Bioluminescence at Multiple Levels in *Vibrio fischeri* ES114', *Journal of bacteriology*, 195: 5051-63.

Septer, A. N., and E. V. Stabb. 2012. 'Coordination of the *arc* regulatory system and pheromone-mediated positive feedback in controlling the *Vibrio fischeri lux* operon', *PLoS One*, 7: e49590.

Stabb, E. V., and E. G. Ruby. 2002. 'RP4-based plasmids for conjugation between *Escherichia coli* and members of the *Vibrionaceae*', *Methods Enzymol*, 358: 413-26.
